# Multi-omics reveals mitochondrial metabolism proteins susceptible for drug discovery in AML

**DOI:** 10.1038/s41375-022-01518-z

**Published:** 2022-02-17

**Authors:** Mika Caplan, Karli J. Wittorf, Kasidy K. Weber, Samantha A. Swenson, Tyler J. Gilbreath, R. Willow Hynes-Smith, Catalina Amador, R. Katherine Hyde, Shannon M. Buckley

**Affiliations:** 1 Department of Genetics, Cell Biology, and Anatomy, University of Nebraska Medical Center, Omaha, NE USA; 2 Fred and Pamela Buffett Cancer Center, University of Nebraska Medical Center, Omaha, NE USA; 3 Department of Pathology and Microbiology, University of Nebraska Medical Center, Omaha, NE USA; 4 Department of Biochemistry and Molecular Biology, University of Nebraska Medical Center, Omaha, NE USA

**Keywords:** Acute myeloid leukaemia, Biochemistry

## Abstract

Acute myeloid leukemia (AML) is a devastating cancer affecting the hematopoietic system. Previous research has relied on RNA sequencing and microarray techniques to study the downstream effects of genomic alterations. While these studies have proven efficacious, they fail to capture the changes that occur at the proteomic level. To interrogate the effect of protein expression alterations in AML, we performed a quantitative mass spectrometry in parallel with RNAseq analysis using AML mouse models. These combined results identified 34 proteins whose expression was upregulated in AML tumors, but strikingly, were unaltered at the transcriptional level. Here we focus on mitochondrial electron transfer proteins ETFA and ETFB. Silencing of *ETFA* and *ETFB* led to increased mitochondrial activity, mitochondrial stress, and apoptosis in AML cells, but had little to no effect on normal human CD34^+^ cells. These studies identify a set of proteins that have not previously been associated with leukemia and may ultimately serve as potential targets for therapeutic manipulation to hinder AML progression and help contribute to our understanding of the disease.

## Introduction

Acute myeloid leukemia (AML) affects the hematopoietic system through abnormal proliferation of myeloid cells in the bone marrow (BM) and ultimately suppresses the production of healthy blood cells. AML is the leading cause of death among adult leukemia patients, although it represents only 35% of those diagnosed [1]. Disease initiation often occurs through recurrent genetic aberrations resulting in the formation of oncogenic fusion proteins [1, 2]. Two genomic alterations found in patients are Mixed Lineage Leukemia (MLL) rearrangements and an inversion of chromosome 16 (Inv(16)) [3–6].

The protein histone-lysine N-methyltransferase 2A (KMT2A), also known as the mixed-lineage leukemia 1 (*MLL1*) gene, contains domains essential for transcriptional activity and chromatin modifications during early hematopoiesis, and fusion proteins generated from each translocation lead to aberrant transcription of MLL target genes [7–11]. *MLL1* has been found to have 80 different fusion partners, although only a few are commonly linked with AML [2]. AML::MLL rearrangements account for 10% of all diagnosed leukemias and these subtypes typically tend to have worse patient prognoses [12–14].

The Inv(16) subtype of AML represents approximately 6% of adults and 9% of pediatric patients [15]. Inv(16) AML results in a fusion between core-binding factor subunit beta (*CBFB*) and myosin heavy chain 11 (*MYH11*) [16, 17]. The resulting fusion protein, CBFβ::SMMHC, through dimerization with the hematopoietic transcription factor RUNX1 leads to a block in myeloid differentiation [16, 18]. Typically, patients diagnosed with this subtype of AML tend to have a more positive prognosis as the cancer is less aggressive for those included in this cytogenetic profile compared to their MLL-rearranged counterparts.

Although multiple advancements in the field have been direct results of the identification and characterization of many AML-initiating mutations, the mechanisms by which the cancer progresses remain ambiguous. While many studies have attempted to investigate these mechanisms through a series of RNA-based methods, these studies have left out potential implications relating to changes that occur at the proteomic level. Our study was designed to specifically interrogate the effect of altered protein expression in AML progression by combining RNA-sequencing (RNA-seq) techniques with a quantitative mass spectrometry (MS) analysis in mouse models of AML: *CBFB::MYH11 (*Inv(16)*)*, *MLL::AF9*, and *MLL::ENL*. These AML translocations were purposefully chosen to highlight the differences in prognosis and potentially identify targets that are differentially or commonly expressed across all translocations. This would allow us to further speculate that targets shared across these AML translocations may also be common among others and could be useful for future therapeutic studies targeting a heterogeneous group of AML translocations. With these combined results, we identified 34 upregulated proteins from AML tumors that remained unaltered at the transcriptional level. These proteins were shown to be associated with mitochondrial function as well as RNA processing. In addition, analysis of adult and pediatric AML patient expression data sets revealed that a number of the proteins differentially expressed had no significant RNA expression alterations. Most notably, we found that mitochondrial proteins ETFA and ETFB were overexpressed in all three translocations and could potentially share a role in the progression of the disease.

AML treatment has remained mostly unchanged for the last few decades, consisting of intensive induction chemotherapy – generally a combination of cytarabine and an anthracycline, such as daunorubicin, with the occasional addition of all trans-retinoic acid (ATRA) for certain subtypes [2, 19–21]. A number of treatments have progressed to clinical trials, but only in recent years have any been approved by the Food and Drug Administration, however, most are limited to specific subtypes of AML and only improves the treatment options for limited subsets of patients [22–25]. Up to 50% of patients treated with intensive induction therapy fail to achieve complete remission and are classified as primary refractory or resistant [26]. These studies identify a set of proteins that have not previously been associated with leukemia and may ultimately serve as potential targets for therapeutic manipulation to hinder AML progression and contribute to our understanding of this disease.

## Materials and methods

### AML mouse models

To induce expression of *Cbfb::MYH11*, 6-8 week-old floxed mice and littermate controls received three intraperitoneal Poly(I:C) injections every other day at a dose of 10 µg/g (Invivogen, San Diego, CA, USA) [27]. *MLL::AF9* mice were purchased from Jackson Laboratory (#009079, Bar Harbor, ME, USA) [28]. To produce *the MLL::ENL* mouse model, Lineage^-^ Sca-1^+^ cKit^+^ (LSK) cells from wild-type (WT) mice were sorted and infected with *MLL::ENL* (Addgene#20873) retrovirus by spinning for 1 h at 3000 RPM. 48 h post-infection LSKs were transplanted with 200,000 WT BM into lethally irradiated WT mice (1000 cGy). Mice were sacrificed when they exhibited signs of disease and/or demonstrated a high white blood cell (WBC) count. cKit/Gr1/CD11b^+^ tumor cells and cKit^+^ WT cells were isolated by positive magnetic selection using Mojo Sort beads following manufacturers protocol (BioLegend).

For transplants of AML cell lines, 5 × 10^5^ MOLM-13 cells were transplanted into sub-lethally (250 cGy) irradiated NOD.Cg-*Prkdc*^*scid*^ *Il2rg*^*tm1Wjl*^/SzJ (NSG) mice (#005557, Jackson Labs). All experiments performed were approved by the Institutional Animal Care and Use Committee of the University of Nebraska Medical Center in accordance with NIH guidelines.

### Flow cytometry analysis

Cells were stained for 1 h on ice with antibodies in 3% FBS in PBS. For apoptosis, staining was performed following BioLegend apoptosis staining protocol and stained with AnnexinV and propidium iodine.

### Patient datasets and samples

*ETFA* and *ETFB* expression and survival analyses in patients utilized data from the publicly available Microarray Innovations in Leukemia (MILE) [29], Therapeutically Applicable Research to Generate Effective Treatments (TARGET) [4], and TGCA Genomic and Epigenomic Landscapes of Adult De Nove Acute Myeloid Leukemia [6] studies. Samples and statistics were previously described [30]. Human CD34^+^ cells were purchased from the Fred Hutch Hematopoietic Cell Procurement and Processing Services Core, and primary AML samples were purchased from Cureline Translational CRO (Supplementary Table 1).

### Cell culture and shRNA lentiviral transduction

HL-60, KASUMI-1, and MOLM-13 cells were purchased and cultured as recommended by ATCC and DSMZ, respectively. All cells were confirmed mycoplasma negative prior to use. Human CD34^+^ cells were cultured in StemSpan SFEM II media with CD34^+^ Expansion Supplement (StemCell Technologies) for 24 h prior to viral infection. The pLKO.1 shRNA plasmid vectors were purchased from Sigma-Aldrich. Lentivirus was produced following manufacturer’s instructions. Cells were infected as previously described and treated with 1 μg/ml of puromycin 48 h post infection. Assays were performed 5 days after initial puromycin treatment. MOLM-13 cells were treated for 72 h with 0.1–50 nM venetoclax and analyzed by MTT assay, and for flow cytometry analysis cells were treated with 5 nM venetoclax for 48 h.

### Immunofluorescence/Western blot

Cells were seeded onto Poly-L-Lysine coverslips (Corning). Cells were fixed at room temp (RT) for 15 min with 4% w/v paraformaldehyde followed by permeabilization and blocking with 3% w/v goat serum and 0.2% v/v Triton X-100 in PBS for 30 min at RT. Cells were stained with primary antibody for 1 h followed by goat anti-rabbit IgG AlexaFluor594 or goat anti-mouse IgG AlexaFluor488 (ThermoFisher Scientific) for 45 min. Confocal imaging was performed using a Zeiss LSM 800 confocal microscope with a 63X/1.4 numerical aperture oil objective.

Western blot analysis was performed as previously described [31]. Antibodies were prepared in 5% BSA in TBST as indicated in supplemental materials and methods.

### RNA isolation, qRT-PCR, and RNA-sequencing

Total RNA was extracted using the Monarch^®^ Total RNA Miniprep Kit (New England Biolabs, Ipswich, MA, USA). cDNA was synthesized using High Capacity RNA-to-cDNA Kit (ThermoFisher) followed by qRT-PCR. Primers in supplemental materials and methods. RNA sequencing was performed by Novogene. RNA-seq analysis was performed by UNMC Bioinformatics Core Facility, and are publicly available on NIH GEO with the dataset identifier GSE193366.

### TMT labeling and mass spectrometry

For global proteome quantification, splenic tumor cells and WT cKit^+^ samples were prepared and TMT-labeled per manufacturer’s protocol (ThermoFisher TMT10plex Mass Tag Labeling Kits), and processed as previously described [31]. The mass spectrometry proteomics data have been deposited to the ProteomeXchange Consortium via the PRIDE partner repository with the dataset identifier PXD025628 [32].

### Mitochondrial respiration

Oxygen consumption rate was assayed using Cell Mito Stress kit (Agilent) and analyzed on Seahorse XF HS mini analyzer.

### Electron microscopy

Cell pellets were fixed in 2% glutaraldehyde, 2% paraformaldehyde in 0.1 M Sorensen’s phosphate buffer, pH7.2. Samples were post fixed in 1% osmium tetroxide in water, dehydrated, and embedded. 60 to 90 nanometer sections were placed on 200 mesh copper grids (Ted Pella). Sections were stained with 2% Uranyl Acetate and Reynolds’ lead citrate for 5 min each step. Sections were examined with a Technai G2 Transmission Electron Microscope (FEI) operated at 80Kv with an AMT camera for digital imaging.

### Statistical analysis

All experiments were performed in triplicate unless noted and statistical analyses were performed using unpaired two-tailed Student’s *t* tests assuming experimental samples of equal variance. Center values represent mean, and error bars depict standard deviation. *p value < 0.05, **p value < 0.01, ***p value < 0.001, ****p value < 0.0001.

### Additional materials and methods

Antibodies, primers, and additional methods are provided in the Supplementary Material.

## Results

### Quantitative mass spectrometry performed in combination with RNA-seq analysis

**Fig. 1:**
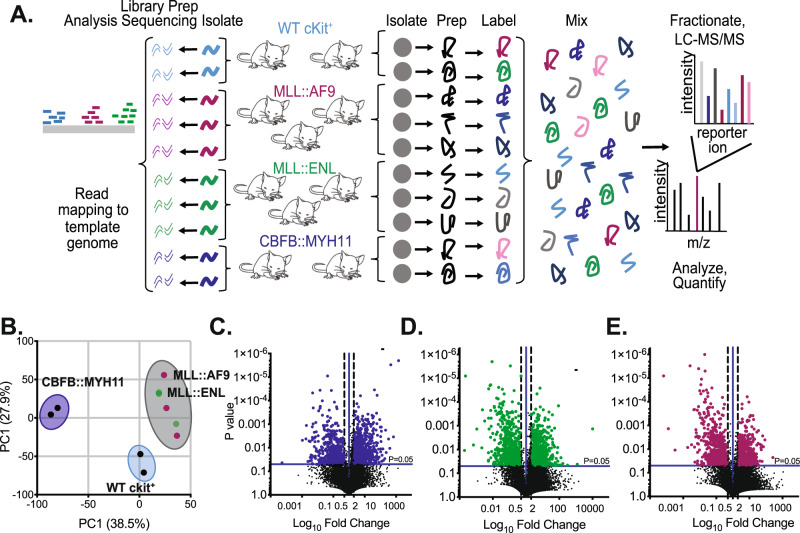
Utilizing AML mouse models to compare proteome vs transcriptome. **A** Preparation schematic for TMT MS and RNA-seq using spleen tumors isolated from mice expressing MLL::AF9 (*n* = 3), MLL::ENL (*n* = 3), CBFB::MYH11 (*n* = 2) and cKit^+^ BM of WT mice (*n* = 2). **B** Principal component analysis of WT cKit^+^ cells compared to MLL::AF9, MLL::ENL, and CBFB::MYH11 tumors. Volcano plots showing fold change of expressed genes from **C** CBFB::MYH11, **D** MLL::ENL, and **E** MLL::AF9 tumors compared to WT cKit^+^ cells.

To compare AML tumors at the transcriptional and translational levels, we utilized 3 different mouse models of AML. To study MLL translocations, we utilized *MLL::AF9* transgenic mice and *MLL::ENL* mice generated by retroviral transduction of the fusion protein into hematopoietic stem and progenitor cells (HSPC) [28, 33]. *Cbfb::MYH11* tumors were generated by crossing *Mx-1 Cre*^*+*^ mice with *Cbfb::MYH11* (*Cbfb*^*+/56M*^) floxed mice, which allows for inducible expression of CBFβ::SMMHC (Inv(16)) following administration of Poly(I:C) [27]. Mouse AML populations are heterogenous and can be similar to immature HSPC which is marked by expression of the cKit receptor [34], but can also be more similar to terminally differentiated myeloid cells (Gr-1^+^/CD11b^+^). WT cKit^+^ BM cells were selected to compare with mouse tumors due to the importance of identifying therapeutic targets that do not affect survival of normal HSPC (Fig. 1A). A total of 10 mice were used for analysis: two WT, three *MLL::AF9*, three *MLL::ENL*, and two *Cbfb::MYH11*. While MLL translocations generally express more mature myeloid cell surface markers such as Gr1 and Mac1 (CD11b), the *Cbfb::MYH11* translocation is characterized by an immature marker, cKit (Supplementary Fig. 1A). Tumor burden within the spleen varied between 40 to 98%, and were purified by positive magnetic selection utilizing anti-cKit, Gr1, and Mac1 (CD11b) antibodies for a more homogenous tumor population (Supplementary Fig. 1A). The 3 different mouse models of AML had similar survival, and all translocations succumbed to disease within the expected period between 20 and 41 weeks (Supplementary Fig. 1B). The mouse models of AML were utilized to perform an RNA-sequencing experiment in parallel with a Tandem Mass Tag (TMT)-labeled mass spectrometry analysis. Proteins from each mouse were isolated for mass spectrometry and in parallel, we isolated RNA from the tumors to prepare for RNA sequencing (Fig. 1A). These initial analyses allowed for the characterization of each sample used in this study to directly compare AML translocations that represented varying outcomes of prognosis.

### RNA-seq identifies differentially expressed genes among AML translocations CBFB::MYH11 (Inv(16)), MLL::AF9, and MLL::ENL

Analysis from RNA-sequencing identified 21,180 expressed genes in the AML mouse tumors and cKit^+^ BM cells. Of these, 3,430 were significantly upregulated or downregulated compared to WT mice. Principal component analysis (PCA) of the genes expressed found that all six of the MLL tumors clustered together, suggesting an overall similarity and lack of distinction across the two MLL translocations, whereas *Cbfb::MYH11* tumors clustered separately from the MLL tumors (Fig. 1B). Differentially expressed genes shared across all three translocations demonstrated that the two MLL translocations shared far more differentially expressed genes (149 and 176; upregulated and downregulated, respectively) than with the *Cbfb::MYH11* translocation (86 and 46; upregulated, 62 and 32; downregulated), suggesting more similarity between these AML subtypes. Although *MLL::AF9* and *MLL::ENL* are both MLL translocations, they only shared 149 upregulated genes in common, suggesting heterogeneity even among MLL tumors (Fig. 1C–E). Taken together, the RNA-seq results identified genes that were differentially expressed within the AML translocations and revealed more similar patterns of RNA expression among MLL translocations.

### TMT-Labeled MS identifies differentially expressed proteins among AML translocations CBFB::MYH11 (Inv(16)), MLL::AF9, and MLL::ENL

**Fig. 2:**
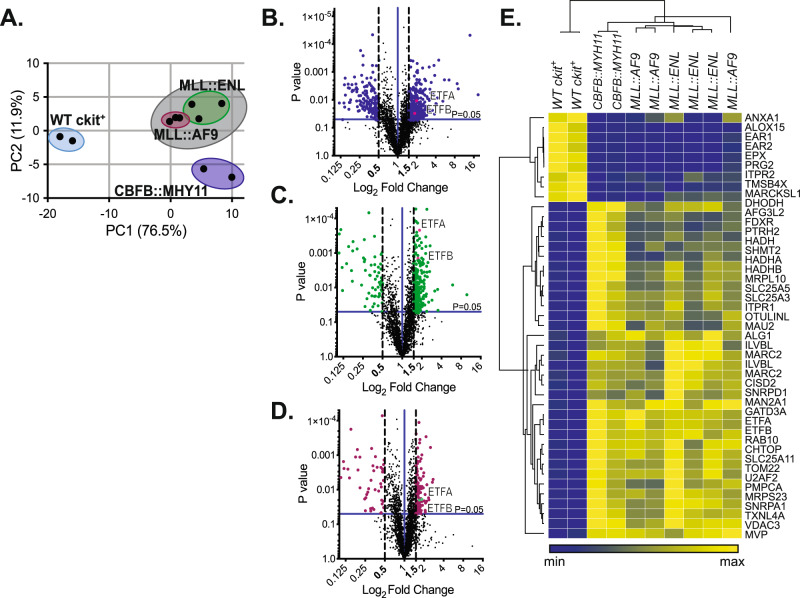
TMT-Labeled MS identifies differentially expressed proteins among AML translocations *CBFB::MYH11*, *MLL::AF9*, and *MLL::ENL*. **A** Principal component analysis of WT cKit^+^ cells compared to *MLL::AF9*, *MLL::ENL*, and *CBFB::MYH11* tumors. Volcano plots showing fold change of expressed proteins from **B** *CBFB::MYH11* (Inv(16)), **C** *MLL::ENL*, and **D** *MLL::AF9* tumors compared to WT cKit^+^ cells. **E** Heatmap with hierarchical clustering of the significantly upregulated (*p* < 0.05, ≥1.5-fold increase over WT) and downregulated (*p* < 0.05, ≤0.5-fold decrease from WT) proteins.

We next evaluated the proteome of the AML mouse tumors. Quantitative mass spectrometry analysis identified 28,708 unique peptides representing 4743 proteins of which 4312 were quantifiable. Only proteins with three or more unique peptides were considered for analysis and of these, 797 were either upregulated or downregulated in at least one of the AML translocations compared to WT. PCA analysis between each translocation found that tumor samples from *MLL::AF9* and *MLL::ENL* clustered separately, indicating a greater level of distinction in expression pattern at the protein level (Fig. 2A). Protein comparison revealed 9 downregulated and 34 upregulated proteins shared among all three translocations, suggesting a greater level of similarity at the protein level for multiple AML translocations (Supplementary Tables 2 and 3). We further compared protein expression of each translocation to WT using volcano plots and found that MLL translocations shared a similar pattern of protein expression when compared to WT for proteins that were upregulated (*p* < 0.05, fold change ≥1.5) or downregulated (*p* < 0.05, fold change ≤0.5) (Fig. 2B–D). Differentially expressed proteins overall clustered by AML translocation (Fig. 2E). These results revealed a contrastingly different pattern of protein expression when comparing each AML translocation as well as identified commonly upregulated and downregulated proteins of interest.

### Comparison of RNA and protein expression reveals differentially expressed proteins shared among data sets

**Fig. 3:**
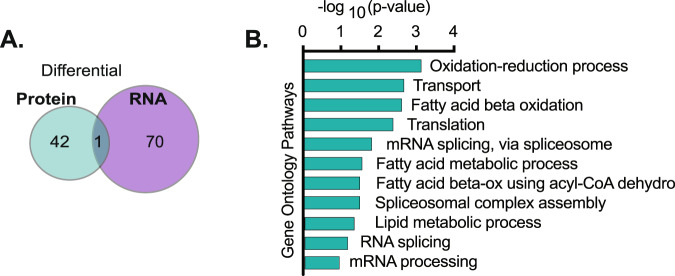
Comparison of RNA and protein expression reveals 42 differentially expressed proteins shared among AML translocations. **A** Combined MS and RNA-seq data comparing overlap of total differentially expressed proteins and genes. **B** Gene ontology analysis showing pathways known to be associated with significantly upregulated (*p* < 0.05, ≥1.3-fold change) proteins using DAVID bioinformatics database.

In order to fully compare between genes and proteins expressed among AML translocations, we compared total number of identified proteins with total number of RNA transcripts and found an overlap of 2,729 proteins. Moreover, we compared total number of differentially expressed proteins with differentially expressed genes and interestingly found that 42 proteins were not differentially expressed at the RNA level (Fig. 3A). Of these 42 differentially expressed proteins, we found that 34 were upregulated and 8 were downregulated in all three translocations. The 34 upregulated proteins were associated with a number of pathways, including RNA splicing and mitochondrial processes (Fig. 3B, Supplementary Tables 2–4) [35]. We further investigated the upregulated proteins identified to examine cellular locations, expression levels, and previously found associations with leukemia (Supplementary Tables 3 and 4). Two novel electron transport proteins: electron transfer flavoprotein subunit α and β (ETFA and ETFB) were significantly overexpressed in tumors from all three AML translocations while their transcripts were unaltered. These findings reveal a set of proteins that are altered distinctly at the proteomic level, with many that are significantly upregulated and participate in key regulatory processes of the cell.

### Electron transfer proteins ETFA and ETFB are differentially expressed as proteins, but are unaltered in RNA-seq and RNA patient data

**Fig. 4:**
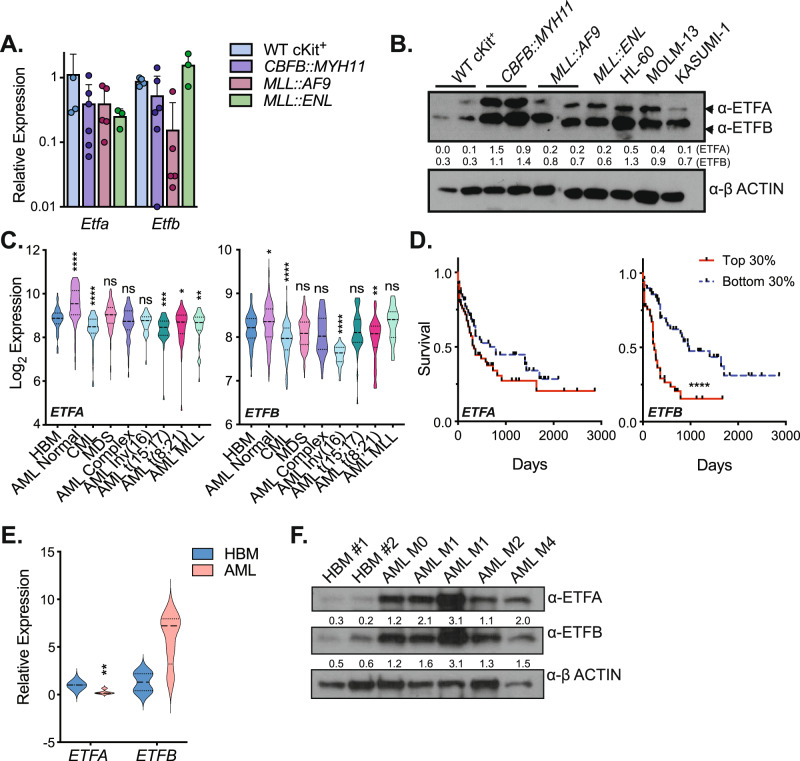
Electron transfer proteins ETFA and ETFB are differentially expressed as proteins but are unaltered as RNA. **A** qRT-PCR for mRNA expression of *Etfa* and *Etfb* in AML mouse tumors (*n* = 3–6). **B** Western blot probing for ETFA and ETFB in mouse AML tumors and in patient-derived AML cell lines compared with mouse cKit^+^ WT BM lysate. **C** *ETFA* and *ETFB* gene expression analysis of patient samples from the MILE study. **D** Overall survival from TCGA AML patient dataset analyzing patients with the highest 30% and lowest 30% of *ETFA* and *ETFB* expression. **E** qRT-PCR and **F** western blot from 2 independent human BM samples and 5 primary patient AML samples (Supplementary Table 1). (*p < 0.05, **p < 0.01, ***p < 0.001, ****p < 0.0001, ns non-significant).

ETFA and ETFB are heterodimeric mitochondrial proteins that form a complex with one flavin adenine dinucleotide (FAD) and one adenosine monophosphate (AMP) [36] to complete the electron transfer flavoprotein (ETF). They are involved in mitochondrial fatty acid and amino acid metabolism by transferring electrons between flavoprotein dehydrogenases. Rare biallelic mutations in *ETFA* and *ETFB* seen in infants to young adults are associated with multiple acyl-CoA dehydrogenase (MADD) deficiencies [37]. MADD is a heterogenous disorder that ranges from infant lethality to late onset with mild phenotype. ETFA and ETFB have not previously been associated with AML; however, AML cells have increased mitochondrial mass and low respiratory chain activity leading them to be sensitive to mitochondrial stress [38]. In addition, the electron transport chain (ETC) complex I has been targeted in AML therapeutics [39, 40]. Validation of RNA and protein expression in mouse tumors confirmed that both ETFA and ETFB show high protein expression but no difference at the RNA level compared to WT cKit^+^ cells (Fig. 4A, B). Patient-derived AML cell lines HL-60, MOLM-13, and KASUMI-1 were compared with cKit^+^ lysate for protein expression of ETFA and ETFB (Fig. 4B). Analysis of patient data from the Leukemia MILE study revealed similar findings to mouse RNA-seq and showed that in adults, most AML subsets have either no significant difference or even significantly decreased gene expression of *ETFA and ETFB* compared to healthy bone marrow (Fig. 4C) [29]. As AML is the second most common childhood leukemia, we utilized the TARGET pediatric study to analyze *ETFA and ETFB* expression and again found down-regulation or no significant change of *ETFA and ETFB* in most subtypes of AML (Supplementary Fig. 2A) [4]. Expression of *ETFA* did not correlate with the probability of patient survival; however, both adult and pediatric patients with high *ETFB* expression had poor survival (Fig. 4D, Supplementary Fig. 2B). A cohort of 5 primary human AML samples ranging from M0-M4 also exhibited low expression of *ETFA* by RNA but high expression at the protein level (Fig. 4E, F). These findings suggest similar patterns of expression of ETFA and ETFB proteins across multiple translocations of human AML and further support the idea that ETFA and ETFB may be potential therapeutic targets in AML.

### Silencing of ETF leads to mitochondrial stress

**Fig. 5:**
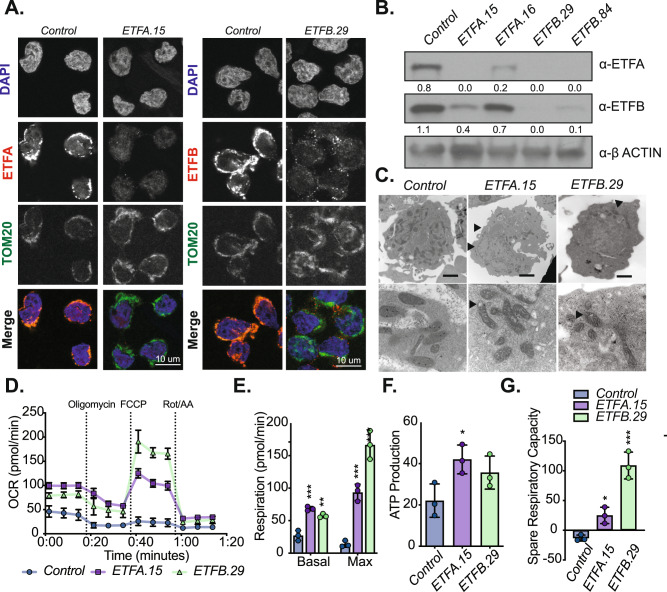
Inhibition of *ETFA* and *ETFB* leads to mitochondrial stress. **A–G** MOLM-13 cells were lentivirally infected with shRNAs targeting *ETFA* and *ETFB* or non-targeting control, and analyzed 72 hours post puromycin selection. Changes in expression analyzed by **A** immunofluorescence with mitochondrial protein, TOM20, and **B** western blot of ETFA and ETFB. **C** Electron microscopy following silencing of *ETFA* and *ETFB*. Analysis of changes in **D** oxygen consumption rate (OCR), **E** basal and maximum respiration rate, **F** ATP production, and **G** spare respiratory capacity by seahorse following knockdown of either *ETFA* or *ETFB*. (*p < 0.05, **p < 0.01, ***p < 0.001, ****p < 0.0001).

Consistent with the localization of mitochondrial protein TOM20 in HELA cells, ETFA and ETFB proteins are localized to mitochondria (Supplementary Fig. 3A, B). In order to determine the role of ETFA and ETFB expression in AML, we utilized shRNA expressing lentivirus in MOLM-13 and HL-60 AML cell lines. MOLM-13 cells have the *MLL::AF9* translocation whereas HL-60 cells do not contain common AML translocations [41–43]. ETFA and ETFB are heterodimers and, interestingly, silencing of either component led to decreased expression at both the RNA and protein level suggesting they may be regulated transcriptionally and post-transcriptionally in a dependent manner (Fig. 5A, B; Supplementary Figs. 3C–E and 4A). Silencing of *ETFA* and *ETFB* in MOLM-13 cells did not alter mitochondrial localization or expression of TOM20 (Fig. 5A). To determine if depletion of *ETFA* altered mitochondrial ultrastructure, we performed electron microscopy in MOLM-13 *ETFA* and *ETFB* knockdown cells. In mitochondria silenced for *ETFA or ETFB*, we observed a high frequency of damaged mitochondria (as indicated by arrows) (Fig. 5C).

We next asked whether knockdown of *ETFA* and *ETFB* alters mitochondrial function. To evaluate mitochondrial metabolism, we performed Seahorse to determine mitochondrial respiration. We found that silencing of *ETFA* and *ETFB* in AML cell lines led to increased oxygen consumption rate (OCR), respiration, extracellular acidification rate (ECAR), ATP production, and spare respiratory capacity (Fig. 5D–G; Supplementary Fig. 4B–G). Taken together, this suggests that AML cell function is sensitive to EFTA and ETFB depletion and inhibition leads to increased mitochondrial metabolism and damages mitochondria.

### AML cells, but not human CD34^+^ normal cells, are sensitive to ETFA and ETFB silencing

**Fig. 6:**
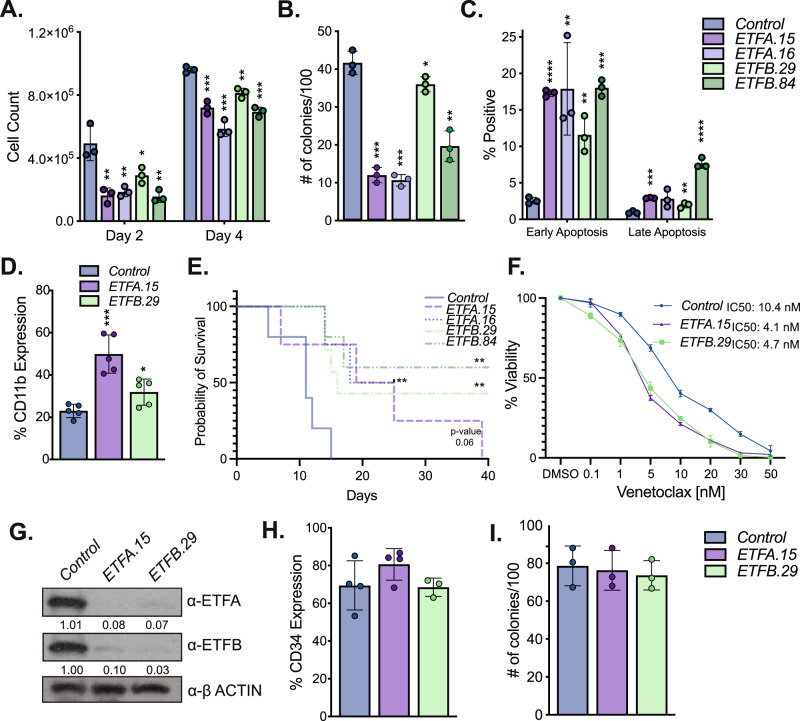
Silencing of *ETFA* and *ETFB* leads to decreased proliferation and survival in AML cells but not in normal human CD34^+^ cells. **A**–**F** Differences between MOLM-13 cells lentivirally infected with shRNAs targeting *ETFA* and *ETFB* or non-targeting control. 72 h post puromycin selection were analyzed by **A** cell counts at day 2 and day 4, **B** ability to form colonies in colony-forming units (CFU) assay, **C** Annexin V and PI staining for percent apoptotic cells, **D** CD11b expression by flow cytometry, and **E** survival rate of sub-lethally irradiated NSG mice transplanted with 5 × 10^5^ MOLM-13 cells infected with shRNAs targeting *ETFA* and *ETFB* or non-targeting control. **F** MTT assay following treatment with 0.1–50 nM venetoclax for 72 h. **G**–**I** Human PB mobilized CD34^+^ cells from 3 independent donors were infected with lentivirus expressing scramble control or shRNAs targeting *ETFA* and *ETFB* and analyzed 72 h post puromycin selection for **G** knockdown by western blot, **H** CD34 expression by flow cytometry, and **I** ability to form colonies in CFU assay (*N* = 3).

To further understand the impact of ETFA and ETFB depletion in AML cell lines, we analyzed cell proliferation, survival, and differentiation. Silencing of *ETFA* and *ETFB* in MOLM-13 and HL-60 cells led to decreased proliferation and ability to generate colony-forming units as well as increased apoptosis and cell death measured by Annexin V and propidium iodine (PI) (Fig. 6A–C; Supplementary Fig. 4H–I). Of note, silencing *ETFA* and *ETFB* in HELA cells did not alter survival of the cells (data not shown). Depletion led to increased expression of Mac1 (CD11b), a marker of mature myeloid cells (Fig. 6D). MOLM-13 cells expressing *ETFA* and *ETFB* shRNA were transplanted into immune-deficient NSG mice (Fig. 6E). Mice transplanted with cells expressing the control shRNA succumbed to AML an average of 10 days post-transplant whereas cells expressing an shRNA targeting *ETFA* and *ETFB* led to mice living significantly longer (Fig. 6E). These studies demonstrate that proliferation and survival of AML cells is dependent on high ETFA and ETFB protein expression. The impact of ETFA and ETFB on proliferation, survival, and mitochondrial stress all contribute to the ability of the cells to engraft, expand and generate disease in the NSG mouse model.

In AML, the B cell lymphoma 2 (BCL-2) protein is highly expressed and plays a role in AML survival by binding pro-apoptotic proteins and inhibiting mitochondrial-induced apoptosis. Currently, venenoclax, a BCL-2 inhibitor has proven efficacious, however some patients develop resistance [44]. To determine whether mitochondrial stress from inhibiting ETFA/B would lead to increased sensitivity to venetoclax, we treated shRNA-targeted MOLM-13 cells with increasing concentrations of venetoclax. Silencing of either *ETFA* or *ETFB* reduced the IC:50 to 4.1 nM and 4.7 nM, respectively, and lead to increased apoptosis (Fig. 6F, Supplemental Fig. 4J). Together, these studies suggest that silencing of* ETFA* and *ETFB* promotes mitochondrial stress leading to increased apoptosis, decreased proliferation, and sensitivity to venetoclax.

AML cells were sensitive to depletion of ETFA and ETFB; however, it is important to understand the impact on normal HSPC. In order to determine the effect of silencing *ETFA* and *ETFB* on human HSPC, we performed knockdown experiments in human CD34^+^ cells isolated from mobilized PB. Similarly to the AML cell lines, we utilized shRNAs targeting *ETFA* and *ETFB*, and cultured the cells in media containing cytokines to promote proliferation, but maintained the CD34^+^ population. We observed similar knockdown efficiency in CD34^+^ cells (Fig. 6G). However, unlike in AML cell lines, silencing *ETFA* and *ETFB* did not lead to differentiation, loss of colony-forming potential, or impacted proliferation (Fig. 6H–I). In addition, we did not see elevated frequency of apoptosis following knockdown, suggesting normal cells are less sensitive to inhibition of ETFA and ETFB (Supplemental Fig. 4K, L). Together these findings suggest that targeting mitochondrial metabolism by interfering with ETFA and ETFB could be a potential therapeutic approach for AML without affecting normal HSPC.

## Discussion

Combining mass spectrometry and RNA-seq analyses in mouse models of AML, we were able to identify a sub-set of proteins that have limited or no alterations in transcript levels compared to their normal counterparts. These upregulated proteins play a role in RNA splicing and mitochondrial processes – two processes currently under investigation for therapeutic targeting and are relied on for AML survival [38, 45–47]. It is believed that leukemic stem cells (LSCs) rely heavily on oxidative phosphorylation (OXPHOS) and metabolic mechanisms of the mitochondria in order to maintain self-renewal and survival [48–50]. Many proteins implicated in these pathways have become novel therapeutic targets and recent studies show that inhibiting parts of mitochondrial metabolism severely impairs the ability of leukemic cells to survive [39, 40, 51]. A recently approved AML drug, venetoclax, targets the mitochondrial protein BCL-2 for inhibition and has seen some success in survival and remission rate; however, many patients have become resistant to venetoclax treatment [52]. Previous research has shown that inhibiting key mitochondrial proteins in venetoclax-resistant AML has relieved some of the acquired resistance, suggesting that targeting additional mitochondrial proteins, including ETFA and ETFB, may be useful as a therapeutic strategy [45]. In our study we find that silencing of *ETFA* and *ETFB* leads to mitochondrial stress, differentiation, apoptosis, and increased sensitivity to venetoclax. These cellular processes combined most likely impede disease initiation and engraftment of the AML cells in NSG mice.

Some of the additional differentially expressed proteins identified from this study correspond with regulators of splicing and spliceosome components. Recently, mutations in genes coding for components of the spliceosome have been found to correlate with certain hematopoietic malignancies, including AML [46, 53–57]. Two predominantly mutated spliceosome proteins (SF3B1 and U2AF1) were both determined to be differentially expressed in the AML models in this study [55, 56, 58–60]. SF3B1 is mutated in a variety of hematopoietic malignancies and these mutations often lead to changes in splice site recognition and splicing components of the protein rather than resulting in a loss-of-function mutation [46, 54, 57]. U2AF1 is another splicing protein specifically required for the recognition of the AG-dinucleotide sequence at the 3’ end of mRNA [59]. Commonly found mutations have led to changes in splicing such as cassette exon skipping/inclusion in targets of the spliceosome, leading to downstream alterations in important cellular pathways. Targeting components of the spliceosome has been one promising area of research in AML, although the mechanisms by which these splicing factors affect hematopoietic malignancies must be further investigated.

Overall, the hits identified from our RNA-seq and MS analyses allow for a continued effort to investigate the roles of these proteins in AML and other hematopoietic malignancies and to understand how they may contribute to the dysregulation of cancer cells. Importantly, silencing of *ETFA* and *ETFB* had minimal impact on survival, maintenance, and differentiation to the HSPC population, suggesting that targeting ETFA and ETFB would not lead to hematopoietic failure. Although further validation and examination is needed, these hits may ultimately serve as potential therapeutic targets in future studies relating to AML.

## Supplementary information

supplemental material
