# Supplementary material for: Multi-omics reveals mitochondrial metabolism proteins susceptible for drug discovery in AML
Source: Leukemia. 2022 Feb 17;36(5):1296–305. doi: 10.1038/s41375-022-01518-z (PMC9061297; doi:10.1038/s41375-022-01518-z)
Supplement: Supplementary file 1 — supplemental material [file 41375_2022_1518_MOESM1_ESM.pdf]

Caplan et al. Supplementary Figure 1

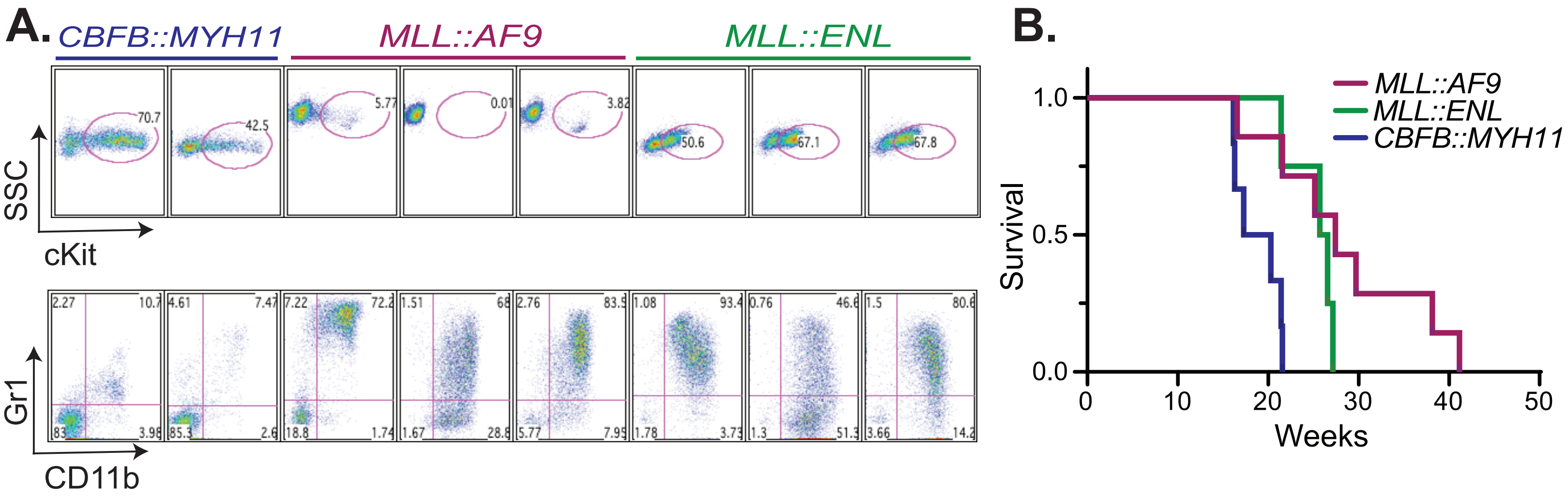

Caplan et al. Supplementary Figure 2

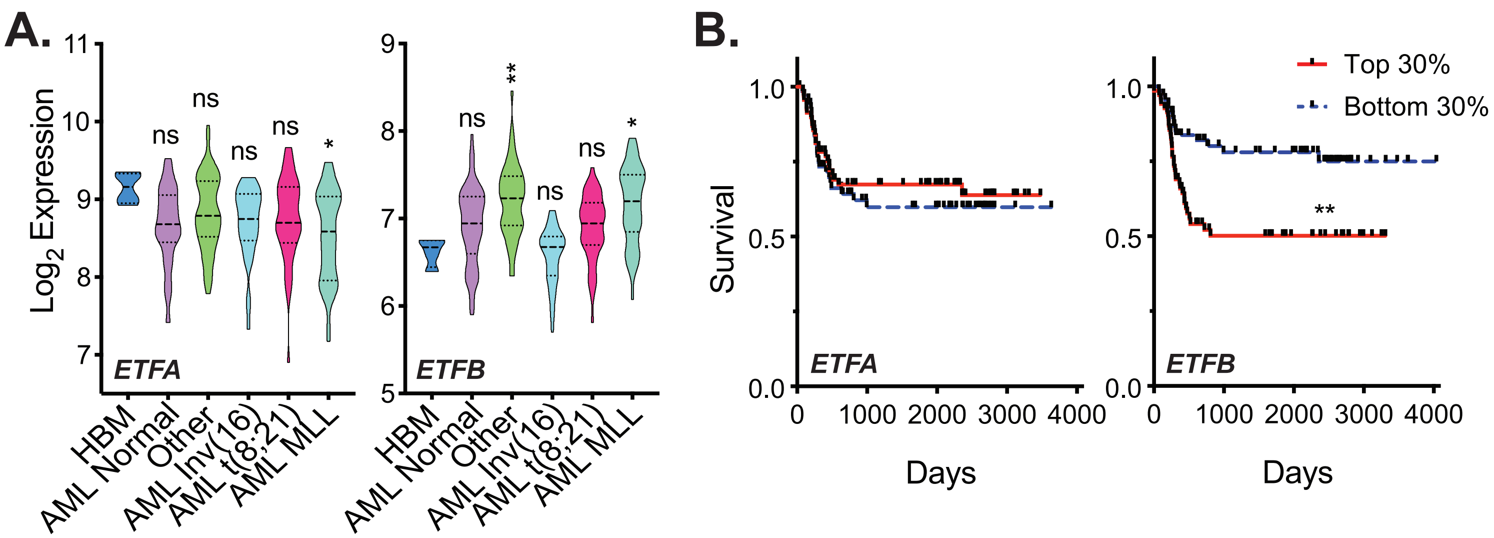

Caplan et al. Supplementary Figure 3

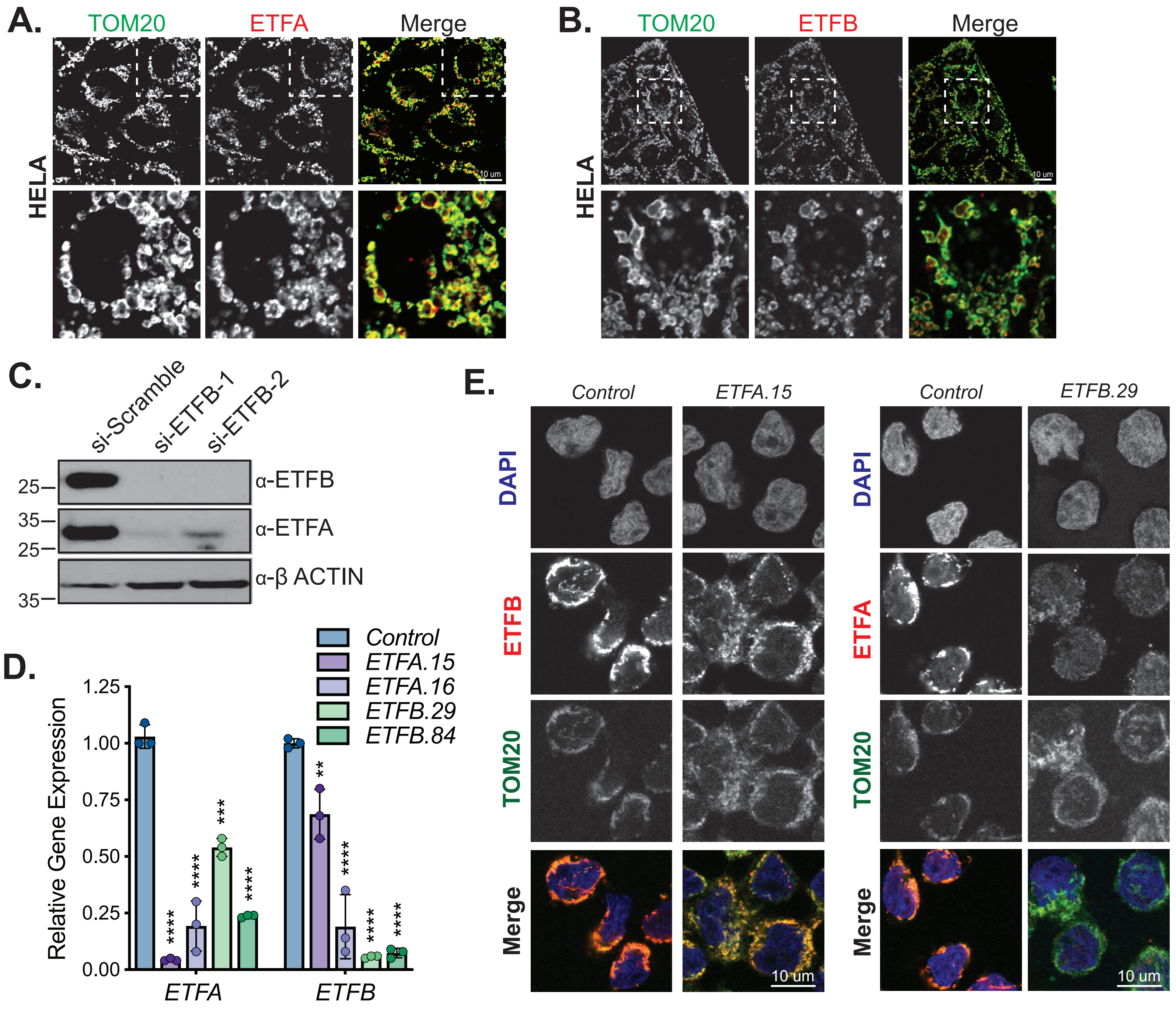

Caplan et al. Supplementary Figure 4

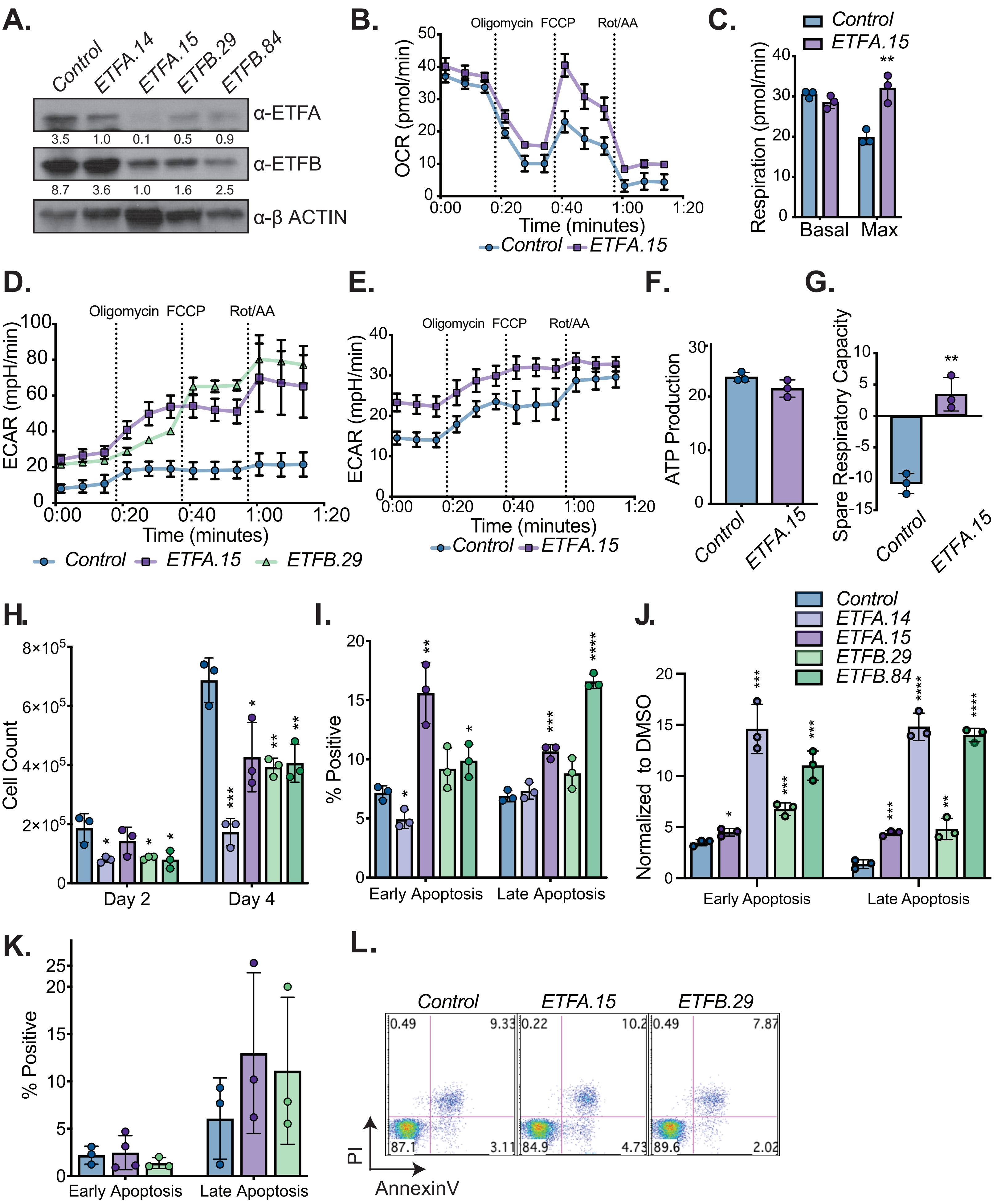

SUPPLEMENTAL MATERIALS AND METHODS

Supplementary Figure 1. Phenotype and expression differences in AML mouse models utilized for proteomic and transcriptomic analysis. A Flow cytometry analysis of cKit, Gr1, and Mac1 (CD11b) cell surface markers on spleen tumor cells from each mouse sample. **B** Kaplan Meyer survival curve of mouse models. Weeks indicated from birth for *MLL::AF9*, from date of transplantation for *MLL::ENL*, and from first Poly:IC injection for *Cbfb::MYH11* (n = 8).

Supplementary Figure 2. Electron transfer proteins ETFA and ETFB expression and survival in pediatric AML. A *ETFA* and *ETFB* gene expression analysis of patient samples from the TARGET study*.* **B** Overall survival from TARGET pediatric AML patient dataset analyzing patients with the highest 30% and lowest 30% of *ETFA* and *ETFB* expression. (*p < 0.05, **p < 0.01, ns = non-significant).

Supplementary Figure 3. ETFA and ETFB are localized within the mitochondria. A-B Immunofluorescence of ETFA, ETFB, and mitochondrial protein, TOM20 in HELA cells. **C** Western blot 48 hours post siRNA transfection in HELA cells. **D** MOLM-13 cells were lentivirally infected with shRNAs targeting *ETFA* and *ETFB* or non-targeting control, and analyzed 72 hours post puromycin selection. Changes in expression analyzed by qRT-PCR. **E** Immunofluorescence of ETFA, ETFB, and mitochondrial protein, TOM20 in MOLM-13 cells 96 hours post lentiviral infection with shRNAs targeting ETFA and ETFB, and 72 hours post puromycin selection.

Supplementary Figure 4. Silencing of ETFA and ETFB leads to increased mitochondrial respiration and apoptosis. HL-60 cells 96 hours post lentiviral infection with shRNAs targeting ETFA and ETFB 72 hours post puromycin selection. Analysis of changes following knockdown via **A** western blot, **B** oxygen consumption rate (OCR), **C** basal and maximum respiration rate, **D** ECAR, **E** ECAR in Molm-13, **F** ATP production, **G** spare respiratory capacity, **H** cell counts at day

2 and day 4, **I** and annexin V and PI staining by flow cytometry. **J** Flowcytometry analysis for annexin V and PI staining for percent apoptotic cells following 48 hrs with 5nM Venetoclax. **K-L** Human PB mobilized $CD34^+$ cells were infected with lentivirus expressing scramble control or shRNAs targeting *ETFA* and *ETFB* and analyzed 72 hours post puromycin selection for percent apoptotic by annexin V and PI flow cytometry staining. (*p < 0.05, **p < 0.01, ***p < 0.001, ****p < 0.0001).

Flow Cytometry Antibodies

**Antibody**	**Fluorochrome**	**Clone**	**Company**
cKit	BV421	2B8	BioLegend
Gr-1	APC	RB6-8C5	BioLegend
Mac1 (CD11b)	PE	M1/70	BioLegend
Annexin V	APC		BioLegend
CD34	FITC	561	BioLegend
CD45	Pacific Blue	2D1	BioLegend

Western Blot and Immunofluorescence Antibodies:

**Antibody**	**Company**	**Ref#**
B-actin-HRP	Santa Cruz	sc-47778
ETFA	Proteintech	12262-1-AP
ETFB	Proteintech	17925-1-AP
TOM20	Santa Cruz	F-10

**qRT-PCR Primer**	**Sequence**
mGapdh F	5’– CAT GGC CTT CCG TGT TCC TA –3’
mGapdh R	5’– CTG GTC CTC AGT GTA GCC CAA –3’
mEtfa F	5'– TGC AGC AAC AAG TGG AGG TA –3'
mEtfa R	5'– AAA GCC GGA CAA GTC TGG AG –3'
mEtfb F	5'– AAA GCC GGA CAA GTC TGG AG –3'
mEtfb R	5'– CAG TTC GGA TGG TCT CCT GG –3'
hGAPDH F	5'– CTT TTG CGT CGC CAG CCG AG –3'
hGAPDH R	5'– CCA GGC GCC CAA TAC GAC CA–3’
hETFA F	5'– GCG GCC TCA TTG CTA CGA TT –3'
hETFA R	5'– GTG CCA CCT TGT CAC ATT TGG –3'
hETFB F	5'– CAT CGA CTA CGC CGT GAA GA –3'
hETFB R	5'– CGG TAC GAA TCG TCT CCT GG –3'

shRNA	Target Sequence
NonTarget Control	CAACAAGATGAAGAGCACCAA
ETFA.64414	GAGAACTATTTATGCAGGAAA
ETFA.64415	GCTTGACCAGAAATTAACAAA
ETFA.64416	GCGGCCTCATTGCTACGATTT
ETFB.291084	CAAGGAGAAGAAGCTGGTGAA
ETFB.64429	GCCCAACATCATGAAAGCCAA

Mass Spectrometry

Samples were loaded onto trap column Acclaim PepMap 100 75 µm x 2 cm C18 LC Columns (Thermo Scientific™) at flow rate of 5 µl/min then separated with a Thermo RSLC Ultimate 3000 (Thermo Scientific™) from 5-20% solvent B (0.1% FA in 80% ACN) from 10-98 minutes at 300 nL/min and 50 ºC with a 120 minutes total run time for fractions one and two. For fractions three to six, solvent B was used at 5-45% for the same duration. Eluted peptides were analyzed by a Thermo Orbitrap Fusion Lumos Tribrid (Thermo Scientific™) mass spectrometer in a data dependent acquisition mode using synchronous precursor selection method. A survey full scan MS (from m/z 375-1500) was acquired in the Orbitrap with a resolution of 120,000. The AGC target for MS2 in iontrap was set as 1 x 10^4 and ion filling time set as 150ms and fragmented using CID fragmentation with 35% normalized collision energy. The AGC target for MS3 in orbitrap was set as 1 x 10^5 and ion filling time set as 200 ms with a scan range of 100-500 and fragmented using HCD with 65% normalized collision energy. Protein identification was performed using proteome discoverer software version 2.2 (Thermo Fisher Scientific) by searching MS/MS data against the UniProt mouse protein database. The search was set up for full tryptic peptides with a maximum of 2 missed cleavage sites. Oxidation, TMT6plex of the amino terminus, GG and GGQ ubiquitination, phosphorylation, and acetylation were included as variable modifications and

carbamidomethylation and TMT6plex of the amino terminus were set as fixed modifications. The precursor mass tolerance threshold was set at 10 ppm for a maximum fragment mass error of 0.6 Da with a minimum peptide length of 6 and a maximum peptide length of 144. The significance threshold of the ion score was calculated based on a false discovery rate calculated using the percolator node. Protein accessions were put into Ingenuity Pathway Analysis (QIAGEN Inc.) to identify gene symbols and localizations. Gene ontology pathway analysis was performed using DAVID Bioinformatics Database 6.8 using the functional annotation tool.

siRNA transfection

HeLa cells were plated on 6 well plates and treated with human ETFA and ETFB siRNA oligonucleotides for 48 h at 37ºC with Lipofectamine RNAiMAX (Thermo Fisher Scientific). The following siRNA oligonucleotides were used in this study: siRNA Universal Negative Control #1 (Sigma-Aldrich, catalog no. SIC001-1NMOL), siRNA ETFA (Sigma-Aldrich, product no. NM_000126, siRNA ID SASI_Hs01_00216824 and SASI_Hs01_00216825), siRNA ETFB (Sigma-Aldrich, product no. NM_001985, siRNA ID SASI_Hs01_00129106).

Supplementary Table 1. AML patient data characteristics.

Sample	Sex	Age at Diagnosis	Stage FAB	Peripheral Blood Blast Count	Diagnosis	Karyotype
1	F	35	M0	36%	De novo	Unknown
2	F	66	M1	91%	De novo	Unknown
3	F	66	M1	95%	De novo	Unknown
4	F	51	M2	50%	De novo	46,XX

5	M	65	M4	94%	De novo	Unknown

Supplementary Table 2. A comprehensive list of overexpressed proteins identified from mass spectrometry reveals RNA and protein expression levels in subtypes *Cbfb-MYH11*, MLL-AF9, and MLL-ENL.

		**Fold Increase protein**			**Fold Increase RNA**		
**Protein Symbol**	**Gene Name**	**Cbfb-MYH11**	**MLL-AF9**	**MLL-ENL**	**Cbfb-MYH11**	**MLL-AF9**	**MLL-ENL**
AFG3L2	AFG3 like matrix AAA peptidase subunit 2	2.31	1.58	1.55	**2.43**	**1.13**	**1.09**
ALG1	ALG1, chitobiosyldiphosphodolichol beta-mannosyltransferase	1.79	1.61	2.31	2.07	2.15	2.61
Chtop	chromatin target of PRMT1	1.93	1.81	1.78	1.44	1.39	1.46
CISD2	CDGSH iron sulfur domain 2	1.75	1.71	2.09	2.07	1.66	1.68
DHODH	dihydroorotate dehydrogenase (quinone)	2.34	1.57	2.01	2.85	1.52	2.42
ETFA	electron transfer flavoprotein subunit alpha	1.99	1.86	1.85	1.67	1.34	1.36
ETFB	electron transfer flavoprotein subunit beta	1.86	1.71	1.75	1.37	0.88	1.7
FDXR	ferredoxin reductase	2.40	1.56	1.51	3.51	1.91	2.1
GATD3A/GATD3B	glutamine amidotransferase like class 1 domain containing 3A	1.84	1.85	1.82			
HADH	hydroxyacyl-CoA dehydrogenase	2.53	1.62	1.61	2.83	1.29	1.35
HADHA	hydroxyacyl-CoA dehydrogenase trifunctional multienzyme complex subunit alpha	2.32	1.63	1.80	2.17	1.41	1.46
HADHB	hydroxyacyl-CoA dehydrogenase trifunctional multienzyme complex subunit beta	2.36	1.62	1.78	1.97	1.05	1.01
ILVBL	ilvB acetolactate synthase like	1.93	1.61	2.12	2.25	1.28	1.9
ITPR1	inositol 1,4,5-trisphosphate receptor type 1	2.03	1.73	1.55	9.7	10.13	5.83
MAN2A1	mannosidase alpha class 2A member 1	1.53	1.59	1.78	3.24	4	3.48
MARC2	mitochondrial amidoxime reducing component 2	2.01	1.78	2.43	1.47	0.99	1.34
MAU2	MAU2 sister chromatid cohesion factor	1.69	1.73	1.86	2.24	2.33	2.08
MRPL10	mitochondrial ribosomal protein L10	2.13	1.54	1.74	1.21	1.49	1.52
MRPS23	mitochondrial ribosomal protein S23	1.75	1.54	1.79	1.47	1.09	1.34
MVP	major vault protein	1.63	1.62	1.55	2.23	2.4	1.73
OTULINL	OTU deubiquitinase with linear linkage specificity like	2.99	2.10	1.75			
PMPCA	peptidase, mitochondrial processing alpha subunit	1.78	1.50	1.72	2.27	1.29	1.35
PTRH2	peptidyl-tRNA hydrolase 2	2.61	1.52	1.73	1.72	0.83	1.04
RAB10	RAB10, member RAS oncogene family	1.78	1.68	1.69	1.87	1.36	0.99
SHMT2	serine hydroxymethyltransferase 2	3.77	1.75	2.16	4.1	1.4	1.68
SLC25A11	solute carrier family 25 member 11	2.19	1.95	2.06	2.02	1.55	1.54
SLC25A3	solute carrier family 25 member 3	2.03	1.60	1.72	1.67	1.09	1.08
SLC25A5	solute carrier family 25 member 5	2.23	1.71	1.86	1.48	1.05	1.08
SNRPA1	small nuclear ribonucleoprotein polypeptide A'	1.83	1.53	1.85	1.57	1.15	1.38
SNRPD1	small nuclear ribonucleoprotein D1 polypeptide	1.76	1.62	1.97	1.3	0.99	1.44

TOMM22	translocase of outer mitochondrial membrane 22	2.20	1.80	2.12	1.18	1	1.12
TXNL4A	thioredoxin like 4A	1.95	1.59	1.82	1.56	0.72	0.52
U2AF2	U2 small nuclear RNA auxiliary factor 2	1.65	1.50	1.67	2.17	1.74	1.66
VDAC3	voltage dependent anion channel 3	1.97	1.67	1.89	1.43	0.8	0.96

Supplementary Table 3. A comprehensive list of overexpressed proteins identified from mass spectrometry reveals cellular localization, related pathways, and associated roles in leukemia.

Protein Symbol	Gene Name	Localization	Pathway	Role in Leukemia
AFG3L2	AFG3 like matrix AAA peptidase subunit 2	Mitochondria	Proteolysis	N/A
ALG1	ALG1, chitobiosyldiphosphodolichol beta-mannosyltransferase	Endoplasmic reticulum	Protein glycosylation	Autophagy protects myeloid cells from oxidative stress, apoptotic signaling
Chtop	chromatin target of PRMT1	Nucleus	mRNA transport/processing	N/A
CISD2	CDGSH iron sulfur domain 2	Endoplasmic reticulum, mitochondria	Energy metabolism	N/A
DHODH	dihydroorotate dehydrogenase (quinone)	Mitochondria	UMP biosynthesis	Inhibitor of DHODH allows for differentiation in MLL AML
ETFA	electron transfer flavoprotein subunit alpha	Mitochondria	Respiratory electron transport	N/A
ETFB	electron transfer flavoprotein subunit beta	Mitochondria	Respiratory electron transport	N/A
FDXR	ferredoxin reductase	Mitochondria	Metabolism	N/A
GATD3A/GATD3B	glutamine amidotransferase like class 1 domain containing 3A	Mitochondria	N/A	N/A
HADH	hydroxyacyl-CoA dehydrogenase	Mitochondria	Beta oxidation	Contributes to embryonic stem cell-like proliferation in MLL-AF9 AML
HADHA	hydroxyacyl-CoA dehydrogenase trifunctional multienzyme complex subunit alpha	Mitochondria	Beta oxidation	Overexpressed in tumor-infiltrating myeloid-derived suppressor cells
HADHB	hydroxyacyl-CoA dehydrogenase trifunctional multienzyme complex subunit beta	Mitochondria, endoplasmic reticulum	Beta oxidation	N/A
ILVBL	ilvB acetolactate synthase like	Membrane	Ion binding	N/A
ITPR1	inositol 1,4,5-trisphosphate receptor type 1	Membrane, endoplasmic reticulum	Signaling	N/A
MAN2A1	mannosidase alpha class 2A member 1	Golgi	Biosynthesis/transport	SNP identified in acute lymphoblastic leukemia and associated with risk
MARC2	mitochondrial amidoxime reducing component 2	Mitochondria, peroxisome	Functionalization of compounds	N/A

MAU2	MAU2 sister chromatid cohesion factor	Nucleus	Cohesin loading onto chromatin	Cohesin complex partners mutated in AML
MRPL10	mitochondrial ribosomal protein L10	Mitochondria	Mitochondrial translation initiation	N/A
MRPS23	mitochondrial ribosomal protein S23	Mitochondria	Mitochondrial translation initiation	N/A
MVP	major vault protein	Nucleus	Protein transport	Correlated with resistance of AML drugs
OTULINL	OTU deubiquitinase with linear linkage specificity like	Nucleus, endoplasmic reticulum	Inactive deubiquitinase	N/A
PMPCA	peptidase, mitochondrial processing alpha subunit	Mitochondria	Protein metabolism	Downregulated in chronic lymphocytic leukemia
PTRH2	peptidyl-tRNA hydrolase 2	Mitochondria	Apoptosis/protein modification	Deletions at locus identified in myeloid malignancies
RAB10	RAB10, member RAS oncogene family	Golgi, cytoplasm, endoplasmic reticulum	Transport	Upregulated by TUG1 in AML cells
SHMT2	serine hydroxymethyltransferase 2	Mitochondria	Metabolism	Identified as Crenolanib target in treatment of AML
SLC25A11	solute carrier family 25 member 11	Mitochondria	Gluconeogenesis	Identified as Crenolanib target in treatment of AML
SLC25A3	solute carrier family 25 member 3	Mitochondria	Transport	Potential biomarker for chronic myeloid leukemia
SLC25A5	solute carrier family 25 member 5	Mitochondria	Transport	N/A
SNRPA1	small nuclear ribonucleoprotein polypeptide A'	Nucleus	mRNA splicing	More abundant in human pluripotent stem cells, interacts with SNRPD1
SNRPD1	small nuclear ribonucleoprotein D1 polypeptide	Nucleus, cytosol	mRNA splicing	More abundant in human pluripotent stem cells, interacts with SNRPA1
TOMM22	translocase of outer mitochondrial membrane 22	Mitochondria	mitochondrial protein import	N/A
TXNL4A	thioredoxin like 4A	Mitochondria	mRNA splicing	N/A
U2AF2	U2 small nuclear RNA auxiliary factor 2	Nucleus	mRNA splicing	Mutations common in myeloid malignancies
VDAC3	voltage dependent anion channel 3	Mitochondria	Transport	N/A

Supplementary Table 4. Corresponding references for identified proteins associated with leukemia.

Protein Symbol	References
AFG3L2	
ALG1	https://www.ncbi.nlm.nih.gov/pubmed/30859901
Chtop	
CISD2	_
DHODH	https://www.ncbi.nlm.nih.gov/pubmed/31253180
ETFA	
ETFB	
FDXR	
GATD3A/GATD3B	
HADH	https://www.ncbi.nlm.nih.gov/pmc/articles/PMC6080909/
HADHA	https://www.ncbi.nlm.nih.gov/pmc/articles/PMC4636942/
HADHB	
ILVBL	
ITPR1	
MAN2A1	https://pubmed.ncbi.nlm.nih.gov/20189245
MARC2	
MAU2	https://www.ncbi.nlm.nih.gov/pmc/articles/PMC4980180/
MRPL10	
MRPS23	
MVP	https://www.ncbi.nlm.nih.gov/pubmed/10025900
OTULINL	
PMPCA	https://www.ncbi.nlm.nih.gov/pmc/articles/PMC3496725/
PTRH2	https://www.ncbi.nlm.nih.gov/pmc/articles/PMC3636093/
RAB10	https://www.ncbi.nlm.nih.gov/pmc/articles/PMC7025684/
SHMT2	https://pubmed.ncbi.nlm.nih.gov/30942519/
SLC25A11	https://pubmed.ncbi.nlm.nih.gov/30942519/
SLC25A3	https://www.ncbi.nlm.nih.gov/pmc/articles/PMC2759651/
SLC25A5	_
SNRPA1	https://www.ncbi.nlm.nih.gov/pubmed/28595116
SNRPD1	https://www.ncbi.nlm.nih.gov/pubmed/28595116
TOMM22	
TXNL4A	
U2AF2	https://www.ncbi.nlm.nih.gov/pmc/articles/PMC6005654/
VDAC3	
